# Supplementary figures and images for: Evolution of the nutritional status and factors associated with undernutrition in children under five of age between 2014 and 2017 in 24 health districts of Burkina Faso
Source: PLOS Glob Public Health. 2025 Jun 5;5(6):e0004645. doi: 10.1371/journal.pgph.0004645 (PMC12140238; doi:10.1371/journal.pgph.0004645)

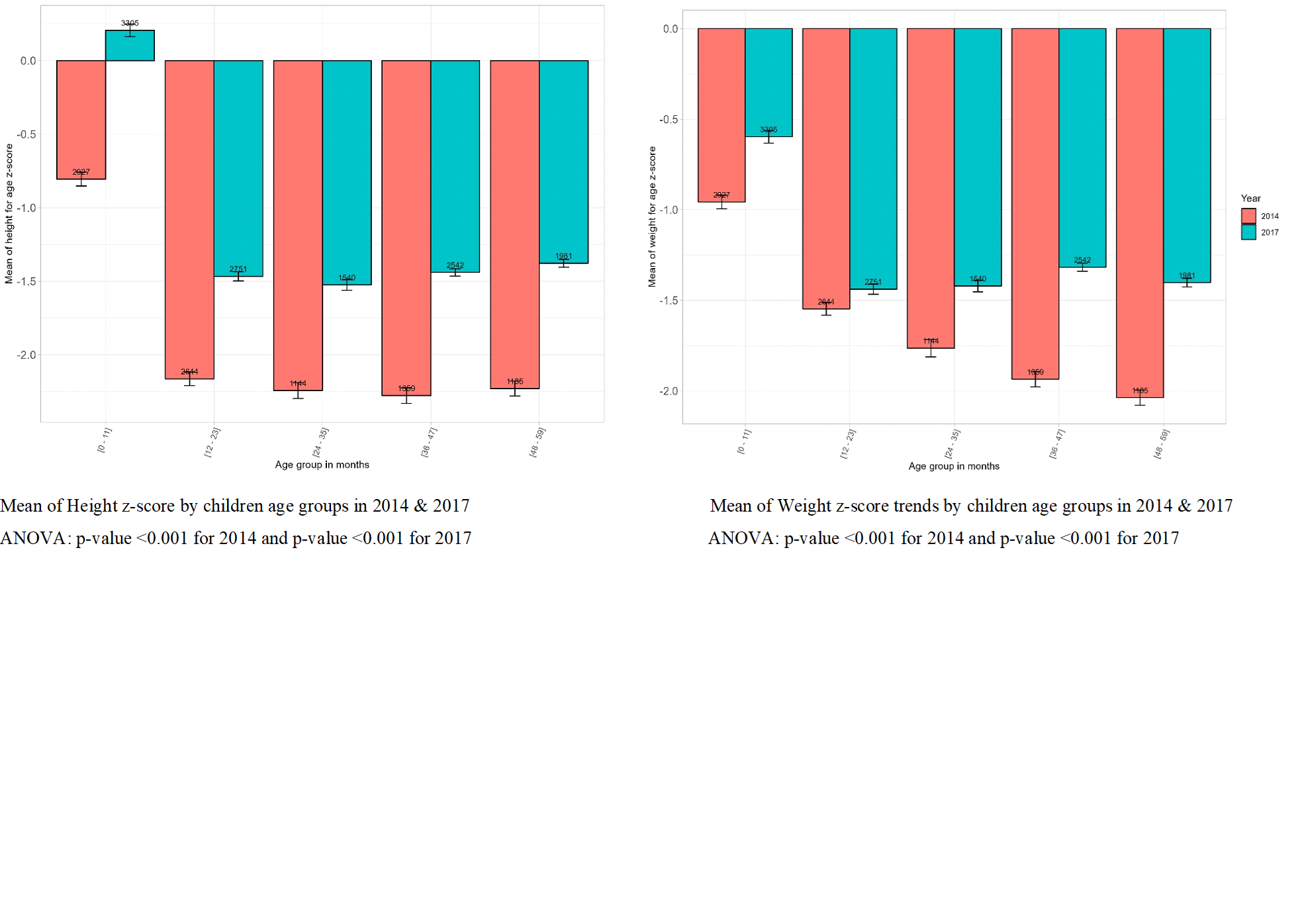

Supplement: S1 Fig — (TIF) [file pgph.0004645.s001.tif]

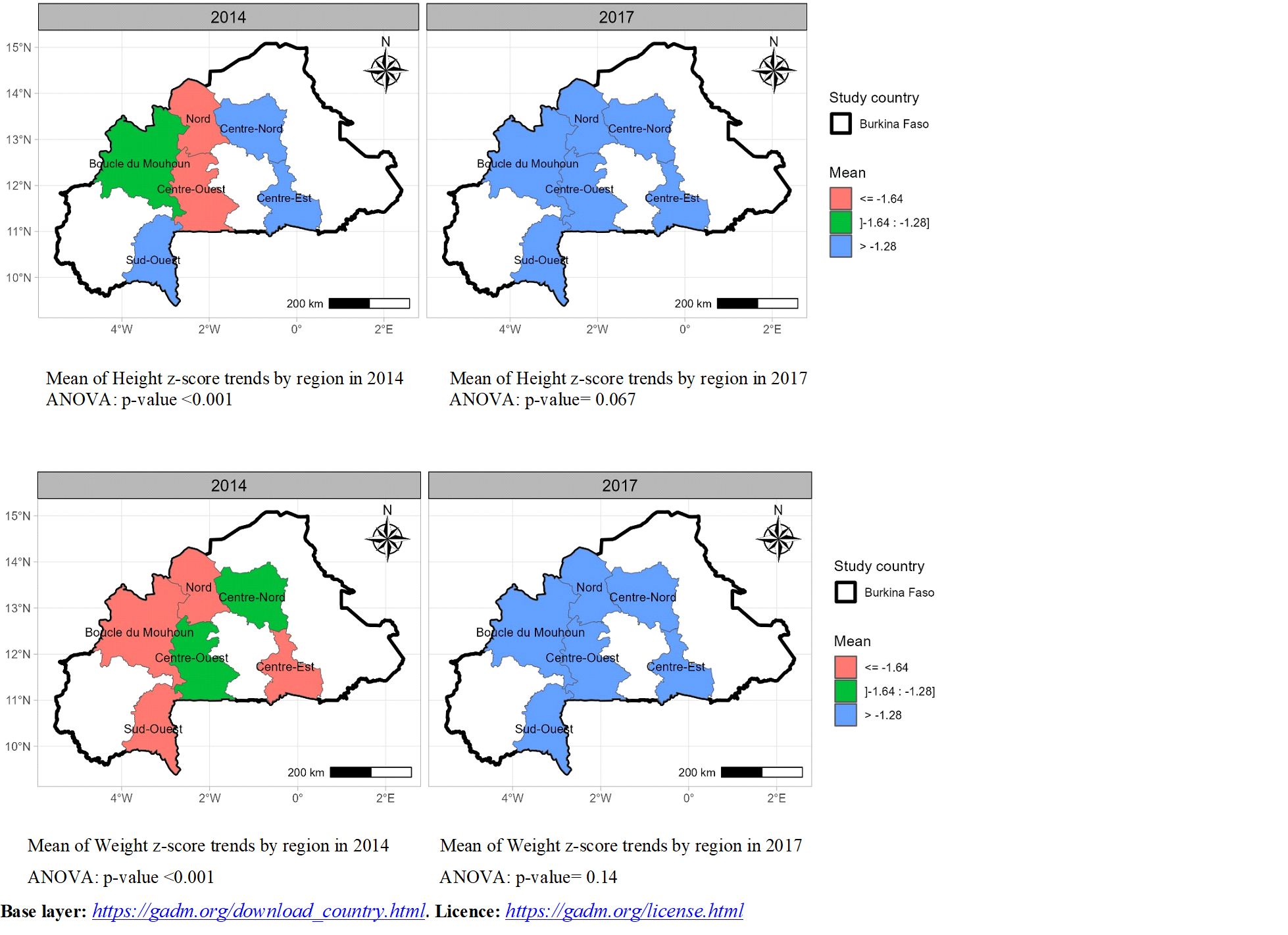

Supplement: S2 Fig — The base layer of this map comes from GADM (https://gadm.org/download_country.html), public domain. This resource is compatible with the CC-BY 4.0 license: https://gadm.org/license.html. (TIF) [file pgph.0004645.s002.tif]
