# Supplementary material for: Evolution of the nutritional status and factors associated with undernutrition in children under five of age between 2014 and 2017 in 24 health districts of Burkina Faso
Source: PLOS Glob Public Health. 2025 Jun 5;5(6):e0004645. doi: 10.1371/journal.pgph.0004645 (PMC12140238; doi:10.1371/journal.pgph.0004645)
Supplement: Attachment — Submitted filename: RESPONSE TO EDITOR_T.Bernadette_PICBOUGOUM.docx [file pgph.0004645.s005.docx]

Tarwendpanga Bernadette PICBOUGOUM	January 2025, 28^th^

Institut National de Santé Publique (INSP) / Centre MURAZ,

2054, Avenue Mamadou KONATE

01 B.P: 390 Bobo-Dioulasso 01 Burkina Faso; [www.centre-muraz.bf](http://www.centre-muraz.bf/)

Phone: (226) 70 34 73 68/ 78 14 43 72

Email : [bernadette.picbougoum@centre-muraz.bf/](mailto:bernadette.picbougoum@centre-muraz.bf/)

[tarwendpanga.picbougoum@uclouvain.be](mailto:tarwendpanga.picbougoum@uclouvain.be) To the Editor of

*PLOS Global Public Health*

Dear Editor

Thank you for your attention to our submission PGPH-D-25-00211 - entitled: **“Evolution of the nutritional status and factors associated with undernutrition in children under five of age between 2014 and 2017 in 24 health districts of Burkina Faso”** and for all the suggestions to complete it.

We have taken all your comments into account in our work and have updated our submission.

**RESPONSE**

We’ve checked your submission and before we can proceed, we need you to address the following issues:

1. Please complete all items on the Human Participants Research Checklist that are relevant for your submission, by following this link:

<http://journals.plos.org/plosone/s/file?id=dc11/PLOSOne_Human_Subjects_Research_Checklist.docx>. There may be overlap between the checklist items and other queries listed here; please address any duplicated queries both in your response email and on the checklist itself. Upload the completed Human Participants Research checklist as file type “Other” when you re-submit your manuscript. This document is for internal journal use only and will not be published if your article is accepted. The requested information will help us to assess whether your submission complies with PLOS’s policies and adheres to applicable reporting standards. Note that your manuscript may be rejected if you provide incomplete or inadequate responses to the checklist questions and that changing the ‘Section/Category’ of your article does not affect this requirement.

*Response*

*Thank you for the suggestion. We have completed the checklist items as following:*

**Human Participants Research Checklist**

***Complete the following if your study involved human participants or human participants’ data. These questions should be addressed for prospective and retrospective studies.***

1. Did you obtain ethics approval for this study?
   - If yes, please upload (file type “Other”) the original approval document you received from your ethics committee. If the original document is in another language, please also provide an English translation.

_YES__ Uploaded	___ N/A

- If you did not obtain ethical approval, please explain why this was not required below.

2. If you prospectively recruited human participants for the study – for example, you conducted a clinical trial, distributed questionnaires, or obtained tissues, data or samples for the purposes of this study, please report in the Methods:
   i. the day, month and year of the **start and end** of the recruitment period for this study.
   ii. whether participants provided informed consent, and if so, what type was obtained (for instance, written or verbal, and if verbal, how it was documented and witnessed). If your study included minors, state whether you obtained consent from parents or guardians. If the need for consent was waived by the ethics committee, please include this information.

___ Completed	_N/A__ N/A

3. If you are reporting a retrospective study of medical records or archived samples, please report in the Methods section:
i. the day, month and year when the data were accessed for research purposes
ii. whether authors had access to information that could identify individual participants during or after data collection

___ Completed	_N/A__ N/A

2. Your study includes child participants, please update your Ethics Statement with confirmation that written informed consent was obtained from the parent/guardian of each participant under 18 years of age.

*Response*

*Thank you for the reminder. We have updated the Ethics Statement in the manuscript with confirmation that written informed consent was obtained from the parent/guardian of each participant under 18 years of age.*

3. You have cited file Fig 3 in the manuscript. However, there are no corresponding files uploaded to the submission/embedded in the manuscript. Please ensure that all files are present.
-Please select 'Figure' from the file inventory for figure files when uploading.
-Please select 'Supporting Information' for supplementary information files.

*Response*

*Thank you for drawing our attention and sorry for omitting it. We have uploaded the file Fig 3 in “ Figure”files.*
