# Supplementary material for: Evolution of the nutritional status and factors associated with undernutrition in children under five of age between 2014 and 2017 in 24 health districts of Burkina Faso
Source: PLOS Glob Public Health. 2025 Jun 5;5(6):e0004645. doi: 10.1371/journal.pgph.0004645 (PMC12140238; doi:10.1371/journal.pgph.0004645)
Supplement: Attachment — Submitted filename: review.docx [file pgph.0004645.s004.docx]

The research work on Evolution of the nutritional status and factors associated 2 with undernutrition in children under five of age between 2014 and 3 2017 in 24 health districts of Burkina Faso has been reviewed and the following comments are as follows

1.Abstract and Introduction Chapter:

- Mention the nutritional status prevailing in the study groups as per WHO data
- Its mentioned thst “From 2014 to 2017, the proportion 38 of severe stunting and severe underweight decreased” mention the reason for it.
- The statement “to assess the evolution of the nutritional status of children 94 under 5 years of age, as well as the factors associated with undernutrition, between 2014 and 95 2017 in Burkina Faso” what do you mean by evolution of nutritional status? Specify the exact aim/objective of the research work.
- Address the Problem statement, research gap, existing scenario clearly in the introduction

2. Materials and Methods

Its rework of the data collected already. what is the method used for analysis? The method used in previous work is different from this? If so, then what is the gap identified?

In Analysis – What is the significance of Z- score? The analysis conducted was based on the significance of what?

How do you conclude the abnormalities are reduced? How do you compare your work with the latest results?

Conclusion:

What are all the modifiable factors identified?

Comment on the reopening this research study now. What significance you have depicted out of your research?
