# Supplementary material for: Evolution of the nutritional status and factors associated with undernutrition in children under five of age between 2014 and 2017 in 24 health districts of Burkina Faso
Source: PLOS Glob Public Health. 2025 Jun 5;5(6):e0004645. doi: 10.1371/journal.pgph.0004645 (PMC12140238; doi:10.1371/journal.pgph.0004645)
Supplement: Attachment — Submitted filename: RESPONSE TO REVIEWERS_T.Bernadette_PICBOUGOUM.docx [file pgph.0004645.s006.docx]

Tarwendpanga Bernadette PICBOUGOUM	April 2025, 28^th^

Institut National de Santé Publique (INSP) / Centre MURAZ,

2054, Avenue Mamadou KONATE

01 B.P: 390 Bobo-Dioulasso 01 Burkina Faso; [www.centre-muraz.bf](http://www.centre-muraz.bf/)

Phone: (226) 70 34 73 68/ 78 14 43 72

Email : [bernadette.picbougoum@centre-muraz.bf/](mailto:bernadette.picbougoum@centre-muraz.bf/)

[tarwendpanga.picbougoum@uclouvain.be](mailto:tarwendpanga.picbougoum@uclouvain.be) To the Editor of

*PLOS Global Public Health*

Dear Reviewers and Editor

Thank you for your attention to our manuscript entitled: **“Evolution of the nutritional status and factors associated with undernutrition in children under five of age between 2014 and 2017 in 24 health districts of Burkina Faso”** and for all the suggestions made to improve it.

We have taken all your comments into account in our work.

**RESPONSE TO REVIEWERS**

PGPH-D-25-00211
Evolution of the nutritional status and factors associated with undernutrition in children under five of age between 2014 and 2017 in 24 health districts of Burkina Faso.

PLOS Global Public Health

Dear Dr. PICBOUGOUM,

Thank you for submitting your manuscript to PLOS Global Public Health. After careful consideration, we feel that it has merit but does not fully meet PLOS Global Public Health’s publication criteria as it currently stands. Therefore, we invite you to submit a revised version of the manuscript that addresses the points raised during the review process.

*Response*

*Thank you for that consideration to our manuscript. We have revised it following the transmitted points raised during the review process.*

Please submit your revised manuscript by May 14, 2025, 11:59 PM. If you will need more time than this to complete your revisions, please reply to this message or contact the journal office at [globalpubhealth@plos.org](mailto:globalpubhealth@plos.org). When you're ready to submit your revision, log on to <https://www.editorialmanager.com/pgph/> and select the 'Submissions Needing Revision' folder to locate your manuscript file.

*Response*

*Thank you for these informations. We submit a revised version of the manuscript with this reply.*

Please include the following items when submitting your revised manuscript:

A rebuttal letter that responds to each point raised by the editor and reviewer(s). You should upload this letter as a separate file labeled 'Response to Reviewers'.

A marked-up copy of your manuscript that highlights changes made to the original version. You should upload this as a separate file labeled 'Revised Manuscript with Track Changes'.

An unmarked version of your revised paper without tracked changes. You should upload this as a separate file labeled 'Manuscript'.

Guidelines for resubmitting your figure files are available below the reviewer comments at the end of this letter.

We look forward to receiving your revised manuscript.

Kind regards,

Hasanain Faisal Ghazi, phd

Academic Editor

PLOS Global Public Health

*Response*

*Thank you for these Guidelines. These have been taken into consideration for re-submission.*

**Journal Requirements:**

1. We have amended your Competing Interest statement to comply with journal style. We kindly ask that you double check the statement and let us know if anything is incorrect.

*Response*

*Thank you for amending. We have double checked the statement “On behalf of all authors, disclose any competing interests that could be perceived to bias this work” and everything is correct.*

2. We do not publish any copyright or trademark symbols that usually accompany proprietary names, eg (R), (C), or TM (e.g. next to drug or reagent names). Please remove all instances of trademark/copyright symbols throughout the text, including ® on page 13.

*Response*

*Thank you for the comment. We have removed the ® from* *Stata® on page 8 instead of Ligne 172, and SE- and LLC.*

3. We have noticed that you have uploaded Supporting Information files, but you have not included a list of legends. Please add a full list of legends for your Supporting Information files after the references list.

*Response*

*Thank you for the suggestion. We have added a list of* *legends for the Supporting Information files after the references on page 21.*

4. Please provide a complete Data Availability Statement in the submission form, ensuring you include all necessary access information or a reason for why you are unable to make your data freely accessible. If your research concerns only data provided within your submission, please write "All data are in the manuscript and/or supporting information files" as your Data Availability Statement.

*Response*

*Thank you for the suggestion. We had already attached the Database. We have provided a complete Data Availability Statement in the submission form*

5. Please review your reference list to ensure that it is complete and correct. If you have cited papers that have been retracted, please include the rationale for doing so in the manuscript text, or remove these references and replace them with relevant current references. Any changes to the reference list should be mentioned in the rebuttal letter that accompanies your revised manuscript. If you need to cite a retracted article, indicate the article’s retracted status in the References list and also include a citation and full reference for the retraction notice.

*Response*

*Thank you for the suggestion. We have updated the references in the manuscript: Lines 72, 74, 79 and 299. We have removed ref 27 (Rapport SMART 2013.pdf.) which was added by mistake. We have also updated the reference links:*

4. Hassfurter K. Global Nutrition Report 2017: Nourishing the SDGs. In: UNICEF DATA [Internet]. 4 Nov 2017 [cited 27 Dec 2024]. Available: https://data.unicef.org/resources/global-nutrition-report-2020/

5. Levels and trends in child malnutrition: Key findings of the 2019 edition | UNICEF. 30 Apr 2019 [cited 27 Dec 2024]. Available: https://www.unicef.org/reports/joint-child-malnutrition-estimates-levels-and-trends-child-malnutrition-2019

7. Burkina Faso : Situation des personnes déplacées internes (PDI) (31 décembre 2022) - Burkina Faso | ReliefWeb. 30 Jan 2023 [cited 23 Dec 2024]. Available: https://reliefweb.int/report/burkina-faso/burkina-faso-situation-des-personnes-deplacees-internes-pdi-31-decembre-2022

8. Enquete_nutritionnelle_nationale_du_burkina_faso_2009.pdf. Available: http://www.unicef.org/bfa/french/enquete_nutritionnelle_nationale_du_burkina_faso_2009.pdf

9. Rapport SMART 2017_Def_fevr_2018.x23411.pdf. Available: https://p4h.world/app/uploads/2023/02/Rapport20SMART202017_Def20fevr202018.x23411.pdf

10. RAPPORT SMART 2014_Final.pdf. Available: http://data.sante.gov.bf/legisante/uploads/6.%20RAPPORT%20SMART%202014_Final.pdf

11. Enquete_nutritionnelle_nationale_du_burkina_faso_2010.pdf. Available: http://www.unicef.org/bfa/french/enquete_nutritionnelle_nationale_du_burkina_faso_2010.pdf

18. BFA 2010 Plan Stratégique Nutrition (2010-2015).pdf. Available: https://extranet.who.int/nutrition/gina/sites/default/files/BFA%202010%20Plan%20Strat%C3%A9gique%20Nutrition%20%282010-2015%29.pdf

39. bfa_rapport_smart_2020_vf.pdf. Available: https://www.humanitarianresponse.info/sites/www.humanitarianresponse.info/files/documents/files/bfa_rapport_smart_2020_vf.pdf

40. Rapport SMART_2022.pdf. Available: https://nutrition.bf/sites/default/files/2023-08/Rapport%20SMART_2022.pdf

41. rapport_smart_rapid_pdi_2023_bfa_15092023.pdf. Available: https://www.nutrition.bf/sites/default/files/2023-10/rapport_smart_rapid_pdi_2023_bfa_15092023.pdf

44. Burkina_Faso_Vulnerability_MAP_FEB_20151.pdf. Available: https://www.usaid.gov/sites/default/files/2022-05/Burkina_Faso_Vulnerability_MAP_FEB_20151.pdf

Additional Editor Comments (if provided):

Please respond to the comments.

*Response*

*Thank you for the suggestion. We have responded to the comments provided.*

[Note: HTML markup is below. Please do not edit.]

Reviewers' comments:

Reviewer's Responses to Questions

**Comments to the Author**

1. Does this manuscript meet PLOS Global Public Health’s [publication criteria](https://track.editorialmanager.com/CL0/https:%2F%2Fjournals.plos.org%2Fglobalpublichealth%2Fs%2Fcriteria-for-publication/1/010f0196330e955f-e7abe190-4547-416d-a14d-7b1098f0306c-000000/MxxjqQ2Z_lE3KdztAuna4FUbvwSiVhTFtXqK9Aw6BpU=206)? Is the manuscript technically sound, and do the data support the conclusions? The manuscript must describe methodologically and ethically rigorous research with conclusions that are appropriately drawn based on the data presented.

Reviewer #1: Yes

Reviewer #2: Yes

2. Has the statistical analysis been performed appropriately and rigorously?

Reviewer #1: No

Reviewer #2: Yes

3. Have the authors made all data underlying the findings in their manuscript fully available (please refer to the Data Availability Statement at the start of the manuscript PDF file)?

The [PLOS Data policy](https://track.editorialmanager.com/CL0/https:%2F%2Fjournals.plos.org%2Fglobalpublichealth%2Fs%2Fdata-availability/1/010f0196330e955f-e7abe190-4547-416d-a14d-7b1098f0306c-000000/y4QoRhfBNPCOBzYNhuY5wo7IJnmr-gexlscQLYv9A8Q=206) requires authors to make all data underlying the findings described in their manuscript fully available without restriction, with rare exception. The data should be provided as part of the manuscript or its supporting information, or deposited to a public repository. For example, in addition to summary statistics, the data points behind means, medians and variance measures should be available. If there are restrictions on publicly sharing data e.g. participant privacy or use of data from a third party—those must be specified.

Reviewer #1: No

Reviewer #2: Yes

4. Is the manuscript presented in an intelligible fashion and written in standard English?

PLOS Global Public Health does not copyedit accepted manuscripts, so the language in submitted articles must be clear, correct, and unambiguous. Any typographical or grammatical errors should be corrected at revision, so please note any specific errors here.

Reviewer #1: Yes

Reviewer #2: Yes

5. Review Comments to the Author

Please use the space provided to explain your answers to the questions above. You may also include additional comments for the author, including concerns about dual publication, research ethics, or publication ethics. (Please upload your review as an attachment if it exceeds 20,000 characters)

Reviewer #1: The research work on Evolution of the nutritional status and factors associated 2 with undernutrition in children under five of age between 2014 and 3 2017 in 24 health districts of Burkina Faso has been reviewed and the following comments are as follows

*Response*

*Thank you for reviewing.*

1.Abstract and Introduction Chapter:

•Mention the nutritional status prevailing in the study groups as per WHO data

*Response*

*Thank you for this comment. We have updated the nutritional status prevailing in the study groups as per WHO data in Abstract Line 37.*

•Its mentioned that “From 2014 to 2017, the proportion 38 of severe stunting and severe underweight decreased” mention the reason for it.

*Response*

*Thank you for this comment. It is a result mentioned in our Abstract with comments mentioning good progress afterwards. We have mentioned the probable reason which is more detailed in the discussion.*

•The statement “to assess the evolution of the nutritional status of children 94 under 5 years of age, as well as the factors associated with undernutrition, between 2014 and 95 2017 in Burkina Faso” what do you mean by evolution of nutritional status? Specify the exact aim/objective of the research work.

*Response*

*Thank you for this comment. This is the objective of our study which means assessing changes in indicators of nutritional status in children under 5 years of age, mainly in terms of undernutrition. We have reworded it as follows: to assess trends in undernutrition among children under 5 years of age, as well as the factors associated with it, between 2014 and 2017 in Burkina Faso.*

•Address the Problem statement, research gap, existing scenario clearly in the introduction

*Response*

*Thank you for the suggestion. We have updated the problem statement, research gap, existing scenario in the introduction Lines 82*

2. Materials and Methods

It’s a rework of the data collected already. What is the method used for analysis? Is The method used in previous work different from this? If so, then what is the gap identified?
In Analysis – What is the significance of Z- score? The analysis conducted was based on the significance of what?

*Response*

*Thank you for this comment and the questioning. We have performed a descriptive analysis of the economic and socio-demographic characteristics of households, children, and mothers in each sample. Pearson’s chi-square test was applied to compare the sample characteristics. We then determined the levels of stunting, wasting, and underweight by calculating the proportion of children classified according to the corresponding z-score, using the total number of children as the denominator. The mean Z-score was then calculated to compare 2014 and 2017, according to the health regions or the age groups of the children using ANOVA. Finally, we used logistic regression (LR) models to assess the associations of stunting and underweight with the explanatory factors in 2017, and for two regions. We compared these factors to those of 2014.*

*The Z-score is a statistical measure that quantifies the distance between a data point and the mean of a dataset. It measures how many standard deviations a data item is from the population mean. A Z-score tells us* ***the number of standard deviations a value is from the mean of a given distribution****. Negative Z-scores indicate the value lies below the mean. Positive Z-scores indicate the value lies above the mean.*

*Our indicators stunting, wasting and underweight were assessed by calculating height-for-age, weight-for-height and weight-for-age indices. Z-scores were used to express the anthropometric standard deviation in line with the Waterlow definitions and the World Health Organization (WHO) 2006 standard.*

*The analysis conducted was based on this classification of indicators. Children were classified as wasted, stunted, or underweight when z-score < -2, moderate when z-score < -2 and -3, and severe when z-score < -3. Weight-for-height z-score also allows the identification of overweight and obesity, but we did not include these indicators in our analysis.*

How do you conclude the abnormalities are reduced? How do you compare your work with the latest results?

*Response*

*Thank you for this comment and the questioning. We have concluded the abnormalities are reduced after comparing the proportion of stunting, underweight and wasting obtained in 2017 with those previously of 2014. We have made subtraction operations, and we have got positive difference meaning decrease. The results are presented in table 2.*

Conclusion:
What are all the modifiable factors identified?

*Response*

*Thank you for this question. The modifiable factors identified were the mother’s educational level, the mother’s occupation, the household economic level and the number of children under five years in the household.*

Comment on the reopening of this research study now. What significance have you depicted out of your research?

*Response*

*Thank you for the suggestion. We would like to note that this research study is a reopening as such, but a continuation of the first one. It is two surveys made in 2014 and 2017. We also aimed to conduct analyses on the second 2017 survey to assess the evolving trends of undernutrition indicators. This is considering the importance of examining recent trends in child nutrition in Burkina Faso, particularly in the face of global public health challenges such as economic crises, pandemics, and especially the context of insecurity that emerged during this period.*

*Our research can be interpreted from several meaningful perspectives: Our findings contribute to the understanding of the factors influencing child malnutrition in Burkina Faso and how this knowledge can support the development of more targeted public health policies; Our results provide a comprehensive overview of the emerging trend shaped by various nutritional programs and interventions. We observed a reduction in undernutrition, which could be seen as an indicator of the success of these programs and interventions; We were able to identify additional factors associated with undernutrition that should be further explored in future research; "We were able to identify additional factors associated with undernutrition that should be further explored in future research, especially in the context of the deteriorating geopolitical environment of the country.*

Reviewer #2: Brilliant manuscript, good writeup and rigorous statistical analysis. However, I have few concerns.

*Response*

*Thank you for reviewing and for the appreciation*

Line 110-111- Kindly clarify what is meant by pre and post implementation assessment, was any intervention carried out? Clearly state so if any.

*Response*

*Thank you for the question and the suggestion. Yes, there was an intervention such as an implementation of the Performance-Based Financing (PBF) program in Burkina Faso. So, to assess the impact of that program, two surveys were planned, first before beginning it and a second one at the end.*

Line 126- Clearly state how malaria infection and anemia were diagnosed among those participants at the household.

*Response*

*Thank you for the suggestion. We have added this in the manuscript Line 126: Malaria infection and anemia were assessed using the Malaria Rapid Diagnostic Test (RDT) and Hemocue test, respectively, were diagnosed in all children under five years of age, pregnant women, and women who had delivered in the last two years at the time of the study, provided they were present in the household during the visit.*

Line 165- The acronym (LR) should immediately follow Logistic regression so that subsequently one can follow when only the acronym is used as in Line 168.
Once these minor concerns are rectified, this is a good article and I recommend it for publishing. Thank you.

*Response*

*Thank you for the suggestion. We have added acronym (LR) Lines 165-166.*

*Thank you for your recommendation.*

6. PLOS authors have the option to publish the peer review history of their article ([what does this mean?](https://track.editorialmanager.com/CL0/https:%2F%2Fjournals.plos.org%2Fglobalpublichealth%2Fs%2Feditorial-and-peer-review-process%23loc-peer-review-history/1/010f0196330e955f-e7abe190-4547-416d-a14d-7b1098f0306c-000000/_jsTld7CyNGVmCNCLhP592zG-Hy2qOHjI_pTsrLvCCg=206)). If published, this will include your full peer review and any attached files.

**Do you want your identity to be public for this peer review?** If you choose “no”, your identity will remain anonymous but your review may still be made public.

For information about this choice, including consent withdrawal, please see our [Privacy Policy](https://track.editorialmanager.com/CL0/https:%2F%2Fwww.plos.org%2Fprivacy-policy/1/010f0196330e955f-e7abe190-4547-416d-a14d-7b1098f0306c-000000/oyGMr7g3RxYOnMBUxvkGszkGgaHEKYZCuyp2e6Yt6K8=206).

Reviewer #1: **Yes:** Hemalatha RJ

Reviewer #2: No

[NOTE: If reviewer comments were submitted as an attachment file, they will be attached to this email and accessible via the submission site. Please log into your account, locate the manuscript record, and check for the action link "View Attachments". If this link does not appear, there are no attachment files.]

While revising your submission, please upload your figure files to the Preflight Analysis and Conversion Engine (PACE) digital diagnostic tool, <https://pacev2.apexcovantage.com/>. PACE helps ensure that figures meet PLOS requirements. To use PACE, you must first register as a user. Registration is free. Then, login and navigate to the UPLOAD tab, where you will find detailed instructions on how to use the tool. If you encounter any issues or have any questions when using PACE, please email PLOS at [figures@plos.org](mailto:figures@plos.org). Please note that Supporting Information files do not need this step.

*Response*

*Thank you for the suggestion. We have uploaded our four figure files to PACE and have gotten PACE adjusted versions.*
